# Verifications of Symptoms

**Published:** 1888-07

**Authors:** Flora A. Waddell

**Affiliations:** At the Homœopathic Medical Society Delaware, Ohio


### VERIFICATIONS OF SYMPTOMS.

(Read by FLORA A. WADDELL, M. D., at the Homœopathic Medical Society Delaware, Ohio, May 8th and 9th, 1888.)

My name being placed in the Bureau of Clinical Medicine for this year, I will respond by calling your attention to a continuation of the subject of last year, the verification of some of the symptoms occurring in my practice since that time. I hope some others present have something to offer on this subject, as *verified* symptoms soon make a strong and indisputable work of reference on which all may depend.

CASE I.—Toes itch and burn; are red and swollen, as if frost-bitten. This condition had annoyed the patient for eight years, every spring and fall; was very severe. The only relief obtained was by applying Chloroform to the affected parts. This would relieve for a short time. Gave Agaricus$^{30x}$ two doses, one hour apart, which entirely relieved for two weeks, when a slight return made it necessary to repeat the dose. Has not had a return since taking the last medicine, over a year ago. Had four other cases of chilblains presenting the same symptoms cured by Agaricus.

CASE II.—A lady having diarrhœa before and during menses, so severe as to debilitate her very much. Had been troubled for the past three years. Noticed, while talking to her, dents on her fingers caused by the scissors she had been using. The patient said any hard pressure left such marks. That made me think of Bovista. On looking over the proving found it had all the symptoms she gave me. Put up some powders of the 30x to be taken one three times per day, during her need of them. The morning of the fifth day she came into the office, saying, "I have something new the matter this month. For the last three mornings I awakened with such fearful cramps in my lower limbs that I cannot stand it any longer. I have to rub and work with them a long time before I can get up, when it passes of until the next morning, when it is the same thing over again." I went to a repertory, as the condition was not a common one, and found Bovista was the remedy, the cramps being a proving. I gave Sac. Lac., and in a few days they disappeared. She was much improved otherwise. The next month gave the 200x, one dose every other night. This did not cause any trouble and cured the diarrhœa, as there has not been a return for the past four months.

Case III.—A lady had pneumonia; was not called until the third day of her sickness. On entering the room the sight was startling. It seemed as if there was small chance for anything to afford relief, still less to cure. The effort for breath was so great, the eyes seemed fairly starting from their sockets. In broken words she gasped, "I cannot *move*, I cannot breathe for this pain in my lungs." Every effort to cough would start the tears. I dissolved a few pellets of Bryonia$^{45m}$ in a teaspoonful of water and dropped a few drops at a time on her tongue. In about fifteen minutes she commenced to breathe easier; in an hour she could turn with help, and in three hours was asleep. Gave Sac. Lac., every hour; only one dose more of Bryonia, same potency, the third day, as improvement seemed to cease. This should convince the most skeptical that virtue exists in the high potency as well as the low when applied according to the rules laid down in our guide book, *The Organon* of Samuel Hahnemann.

Case IV.—A Methodist minister's wife had for the last eight years been afflicted with rush of blood to the head, chest, and arms, so severe as to make life a misery. Feet cold, and must constantly move them, especially after lying down at night. Hering gives the symptoms under Lilium tig. like this: Fullness of head with pressure outward, as if contents would be forced through every aperture, conscious pulsations over whole body, and out pressing in hands and arms, as if the blood would burst through the vessels. Constant motion of the feet and limbs on lying down. Gave Lilium$^{200x}$, one dose every night until relieved, then three times per week until cured. The peculiar symptom in this case was that she paid her bill and sent a number of her friends to me for treatment. In our part of the country the ministers expect us to do their work for thanks and help to pay their salaries besides.

I now wish to call the attention of the physicians present to a remedy called Crawley. I have never seen it mentioned in homœopathic literature, have never known of a proving. I have produced a few symptoms in cases where I have been giving it, but they were too sick at the time to push the drug effects any further. King's *Dispensatory* gives all the knowledge I have of it except my own experience. It states that it was used by the M. D.'s of long ago for fevers in its powdered state. I obtained some fresh roots and made a tincture of them. The high price I paid for them should have made it resemble Aurum metallicum. From this I prepared the third potency and commenced experimenting with it. I found it would cure the common fevers for which we usually give Aconite very much the same way. It is very effective in the first stage of typhoid, which Aconite is not. It will break *hectic* fever where it has not passed beyond the stage of cure without leaving the patient prostrate afterward. There is another peculiar fever we meet with occasionally that I have never been able to cure with anything. Perhaps others have been more successful. Such cases as have had this condition for a number of years coming on in the forenoon and lasting until midnight, the fever reaching its height about two o'clock in the afternoon. Can bear only the lightest covering at night, even in the winter time. They do not have a chill in the beginning, no perspiration following. From twelve o'clock until morning can be covered and sleep well. Appetite good. Usually able to be about, but very nervous in the afternoon.

This fever has some diseased conditions aside from the fever not the same in all cases. I have found it in both sexes, and with very different symptoms in each case. Would remove all the rest of the trouble with the properly selected remedy, and expect this fever to go also. Alas for my expectations! they failed to be realized. Without a knowledge of this remedy should have failed in curing my cases. As an illustration, I will give one case. Lady, thirty years of age. Has had urinary trouble for five years, accompanied with headache, etc., a well marked Sulphur case. Gave Sulphur$^{200x}$ and upward until it removed all the urinary and head symptoms. She said: "There is one trouble your medicine does not reach. I am better than I ever expected to be, and would think I was well if it was not for this fever I have coming on every day in the forenoon and lasting till midnight. I cannot bear only the lightest covering, and the soles of my feet, the inside of my hands, feel as if on fire, and I get so nervous in the afternoon I scarcely know what to do. Still can work some, and go about all the time." I said, "How long have you had this trouble so bad?" She answered, "About four years." I had given Sulphur. Now gave Sanguinaria, then tried Chamomilla, but without improvement. Then I gave third potency of Crawley root, a dose to be taken three times a day. In three weeks was well. My hearers are ready to ask by this time, "Did you never have any failures? Do you want us to think your judgment is never at fault, that your medicine always works like a charm?" I would say this. It is ten years since I commenced to practice Homœopathy according to the light I had given me. I have cases all the time in which my diagnosis is at fault. Sometimes from carelessness, sometimes from my imperfect knowledge of the materia medica, and, to tell the truth about it, sometimes because I do not know. I would relate some of it if I could see where it could benefit you, but I do not doubt but that you all have had enough of that in your own early experience not to need any of mine in addition; but this I do know, my greatest successes are achieved with the single remedy and potencies of the 200x and upward. Wishing you all success and happiness, I bid you adieu.

